# Coracohumeral Ligament Sectioning in Teres Major versus Latissimus Dorsi Tendon Transfer in Brachial Plexus Birth Palsy

**DOI:** 10.1055/a-2618-3151

**Published:** 2025-06-19

**Authors:** Javier Gutierrez-Pereira, Antonio Garcia-Lopez

**Affiliations:** 1 Spanish National Reference Center for Brachial Plexus Surgery, Hospital General Universitario de Alicante: Hospital General Universitario Dr Balmis, Alicante, Spain

**Keywords:** coracohumeral ligament, latissimus dorsi, brachial plexus birth injury, tendon transfer, teres major

## Abstract

**Background:**

The latissimus dorsi tendon transfer (LDTT) to the supraspinatus tendon is a common procedure for restoring shoulder abduction and external rotation in upper root brachial plexus lesions. However, its association with scapular retraction often limits shoulder abduction.

**Methods:**

This retrospective study compared the functional outcomes of teres major tendon transfer (TMTT) combined with anterior coracohumeral ligament release (CHLR) versus LDTT. Patients who underwent surgery at our center between January 2012 and December 2022 were included, with a mean follow-up of 38 months. Outcomes were assessed using a range of motion and the Mallet scale.

**Results:**

A total of 40 patients were included, with 20 undergoing TMTT with CHLR and 20 undergoing LDTT. The overall mean age was 3.9 years (range: 2.7–4.8), with mean ages of 3.4 years (range: 2.2–5.2) in the LDTT group and 4.1 years (range: 2.8–5.2) in the TMTT with the CHLR group. The TMTT with CHLR group achieved mean gains of +77 degrees in active abduction, +44 degrees in active external rotation, and +46 degrees in passive external rotation. In comparison, the LDTT group demonstrated gains of +46, +27, and +24 degrees, respectively, for the same parameters.

**Conclusion:**

TMTT combined with anterior CHLR significantly improves shoulder abduction and external rotation in patients with Brachial plexus birth injury, particularly those with internal rotation contractures. This technique offers superior functional outcomes compared to LDTT, suggesting a more effective therapeutic alternative.

**Level of Evidence:**

IV, retrospective comparative study.

## Background

One of the primary challenges associated with brachial plexus birth injury (BPBI) affecting the upper trunk is the imbalanced forces between the external and internal rotator cuff muscles. This imbalance often results in internal rotation deformity, leading to limitations in shoulder abduction and external rotation, which tends to worsen with growth.

Traditionally, the restoration of this balance has involved the use of a latissimus dorsi tendon transfer (LDTT) to the supraspinatus tendon, a technique initially described by L'Episcopo and later modified by Hoffer, who incorporated the teres major (TM). However, a prevalent issue in most cases involves subscapularis muscle contracture, anterior coracohumeral ligament retraction, and TM muscle contracture collectively leading to scapula retraction and limited abduction. To address this, we have proposed the approach of TM tendon transfer (TMTT) to the supraspinatus, combined with anterior coracohumeral ligament release (CHLR).

This study aimed to compare the functional outcomes of TMTT with CHLR versus the conventional LDTT approach to elucidate the potential superiority of our proposed technique over the classical method.

## Material and Methods

We conducted a retrospective observational cohort study to analyze 40 patients with BPBI who underwent surgery at our center between January 2012 and December 2022. All surgeries were performed by the same senior surgeon. Of the total 40 patients, 20 underwent LDTT (14 males and 6 females), while the remaining 20 underwent TMTT with CHLR (11 males and 9 females). Of the cases, 24 presented with right-sided lesions, and 16 with left-sided lesions. In all patients, elbow and hand function were preserved.

The series comprised children diagnosed with BPBI characterized by upper root involvement and aged 2 to 5 years. The procedure was indicated for patients exhibiting upper brachial plexus root injury, persistent weakness in shoulder abduction and external rotation, internal rotation contracture, and mild or moderate glenohumeral dysplasia (types II, III, and IV of the Waters et al Classification 1 ). All patients in the study demonstrated functional TM and LD muscles. Exclusion criteria encompassed children below 2 years and above 5 years of age, as well as those with complete brachial plexus involvement. Informed consent for the surgical intervention was obtained from the legal representatives of all patients. This research project received approval from the Ethics and Research Committee of our center.

### Functional Assessment

Shoulder function was evaluated preoperatively and postoperatively, considering the range of motion (ROM) for active abduction, active external rotation in abduction, and passive external rotation in adduction. Additionally, the Mallet scale, 2 consisting of five parameters (abduction, external rotation, ability to bring the hand to the mouth, ability to bring the hand to the nape of the neck, and internal rotation) was employed for evaluation. Follow-up assessments were conducted at 6 weeks and 6 months postsurgery.

### Surgical Technique of TMTT Associated with CHLR

**Fig. 1:**
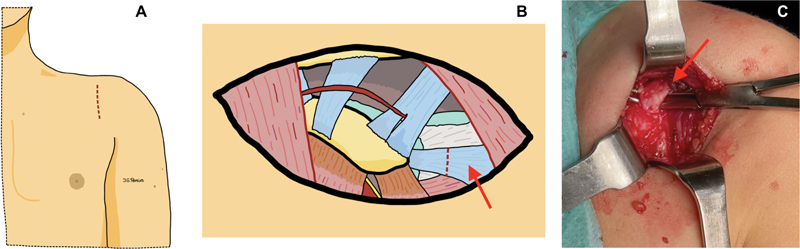
Author's illustrations. ( **A** ) Deltopectoral approach to the shoulder centered on the coracoid process. ( **B** ) The red dashed line indicates the release of the anterior coracohumeral ligament. ( **C** ) Surgical image showing the anterior coracohumeral ligament ready for release.

The surgical procedure involves two approaches, requiring the patient to be positioned in lateral decubitus with the arm adequately covered to facilitate unrestricted movement during the operation. Initially, a limited deltopectoral approach of 3 to 4 cm is undertaken. The incision starts one centimeter below the clavicle, extending over the coracoid process and progressing toward the axilla. Dissection is performed by navigating through the planes, delving into the muscle plane between the deltoid and pectoralis major muscles while preserving the cephalic vein. Then, the anterior coracohumeral ligament, a structure frequently exhibiting hypertrophy in these patients, is identified and released. External rotation of the shoulder is then performed, confirming its improvement ( Fig. 1 ).

Subsequently, a posterior shoulder approach is undertaken, commencing from the acromion and extending toward the posterior axillary fold. From there, the direction is adjusted toward the inferior angle of the scapula, following the direction of the lateral border of the scapula, centered on the TM until its humeral insertion is exposed. The tendon of the TM is then identified and dissected, and a precise cut is made as close as possible to its insertion in the intertubercular groove of the humerus.

**Fig. 2:**
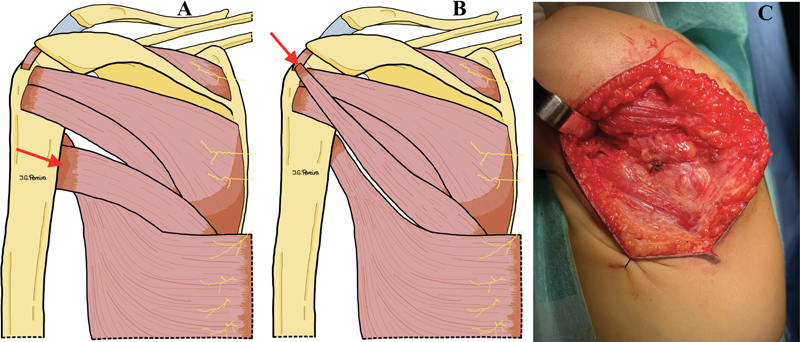
Author's illustrations of the surgical technique, posterior anatomical view of the humeral region. ( **A** ) The teres major tendon is inserted together with the latissimus dorsi tendon into the intertubercular groove of the humerus. ( **B** ) Depiction of the transfer of the teres major tendon to the supraspinatus muscle. ( **C** ) Surgical image of the final result of the transfer.

To expose the tendons of the supraspinatus and infraspinatus muscles, the posterior deltoid muscle is detached cranially. Subsequently, the tendon of the TM is transferred to the tendon of the supraspinatus within the rotator cuff and secured with an absorbable suture ( Fig. 2 ). The transfer is conducted beneath the cranially detached deltoid muscle. This technique effectively transforms the TM into an abductor and external rotator of the shoulder. Finally, a drainage tube is placed, and closure is performed in layers.

**Fig. 3:**
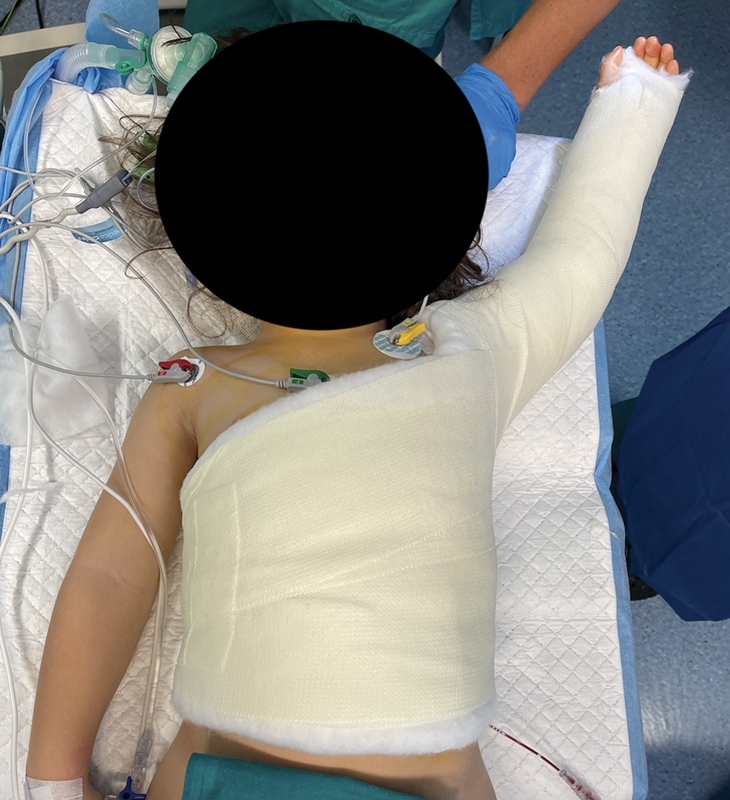
Arm and trunk immobilization in abduction and external rotation.

Finally, an arm and trunk immobilization is applied, which the patient is required to wear for 6 weeks ( Fig. 3 ). Upon removal, a physical therapy protocol is initiated, focusing on the restoration of normal shoulder ROM and the enhancement of shoulder abduction and external rotation. In the LDTT group, we implement identical immobilization measures and adhere to the same rehabilitation protocol.

## Results

The overall mean age of the cohort was 3.9 years (range: 2.7–4.8). Within this, the mean age for the LDTT group was 3.4 years (range: 2.2–5.2), and the mean age for the TMTT with the CHLR group was 4.1 years (range: 2.8–5.2). Following a mean follow-up of 38 months (range: 12–120), a significant improvement in global shoulder function was observed in all patients who underwent surgery, with superior functional outcomes in the TMTT with the CHLR group.

In the TMTT with CHLR group, the mean preoperative abduction was 94 degrees, which significantly improved to 171 degrees postoperatively (+77 degrees). Likewise, the mean preoperative active external rotation increased from 42 to 86 degrees postoperatively (+44 degrees). Additionally, the mean preoperative passive external rotation showed improvement, increasing from 22 to 68 degrees postoperatively (+46 degrees).

In the LDTT group, the mean preoperative abduction was 112 degrees, which also increased postoperatively to 158 degrees (+46 degrees). Similarly, the mean preoperative active external rotation increased from 31 to 58 degrees postoperatively (+27 degrees). Additionally, the mean preoperative passive external rotation increased from 15 to 49 degrees postoperatively (+34 degrees; Table 1 ).

**Table 1:** Mean preoperative and postoperative mobility grades of both groups

Range of motion	TMTT + CHLR ( *n* = 20)		LDTT ( *n* = 20)	
	Preoperative	Postoperative	Preoperative	Postoperative
Active abduction	94 degrees (range: 70–120)	171 degrees (range: 90–180)	112 degrees (range: 40–150)	158 degrees (range: 85–170)
Active external rotation (in abduction)	42 degrees (range: 20–90)	86 degrees (range: 70–90)	31 degrees (range: 0–60)	58 degrees (range: 25–85)
Passive external rotation (in adduction)	22 degrees (range: 0–70)	68 degrees (range: 45–80)	15 degrees (range: −10 to 50)	49 degrees (range: 10–90)

Abbreviations: CHLR, Coracohumeral ligament release; LDTT, Latissimus dorsi tendon transfer; TMTT, Teres major tendon transfer.

According to the Mallet scale ( Table 2 ), the mean abduction was 3.2 ± 0.5 preoperatively and 3.8 ± 0.5 postoperatively in the TMTT with CHLR group, while in the LDTT group, it was 3.3 ± 0.6 preoperatively and 3.8 ± 0.5 postoperatively. The mean external rotation measured 2.6 ± 0.4 preoperatively and 3.6 ± 0.6 postoperatively in the TMTT with CHLR group and 2.4 ± 0.8 preoperatively and 3.3 ± 0.4 postoperatively in the LDTT group. The mean hand-mouth movement in the TMTT with CHLR group was 2.3 ± 0.4 preoperatively and 3.1 ± 0.5 postoperatively, while in the LDTT group, it was 2.3 ± 0.6 preoperatively and 3.0 ± 0.7 postoperatively. The mean hand-neck movement in the TMTT with CHLR group was 2.7 ± 0.6 preoperatively and 3.5 ± 0.7 postoperatively, while in the LDTT group, it was 2.8 ± 0.5 preoperatively and 3.4 ± 0.7 postoperatively. Finally, the mean internal rotation (hand-back) in the TMTT with CHLR group was 2.7 ± 0.4 preoperatively and 2.7 ± 0.5 postoperatively, while the LDTT group harbored a mean of 2.8 ± 0.7 preoperatively and 2.9 ± 0.6 postoperatively.

**Table 2:** Comparative preoperative and postoperative Mallet scores of both groups

Mallet score	TMTT + CHLR ( *n* = 20)		LDTT ( *n* = 20)	
	Preoperative	Postoperative	Preoperative	Postoperative
Active abduction	3.2 ± 0.5	3.8 ± 0.5	3.3 ± 0.6	3.8 ± 0.5
External rotation	2.6 ± 0.4	3.6 ± 0.6	2.4 ± 0.8	3.3 ± 0.4
Hand to mouth	2.3 ± 0.4	3.1 ± 0.5	2.3 ± 0.6	3.0 ± 0.7
Hand to neck	2.7 ± 0.6	3.5 ± 0.7	2.8 ± 0.5	3.4 ± 0.7
Hand to spine	2.7 ± 0.4	2.7 ± 0.5	2.8 ± 0.7	2.9 ± 0.6

Abbreviations: CHLR, coracohumeral ligament release; LDTT, latissimus dorsi tendon transfer; TMTT, teres major tendon transfer.

## Discussion

When comparing active abduction between the two groups, a significant improvement of 77 degrees was observed in the TMTT with the CHLR group, while the LDTT group showed an improvement of 46 degrees. Patients in the TMTT with CHLR group initially exhibited a lower preoperative function than the LDTT group (94 degrees vs. 112 degrees) but achieved superior postoperative outcomes. In the case of active external rotation in abduction, there was an improvement of 44 degrees in the TMTT with the CHLR group compared to the 27 degrees improvement in the LDTT group. Importantly, the LDTT group started with a lower preoperative function (31 degrees vs. 42 degrees), and the postoperative mobility result reflected a substantial difference (58 degrees vs. 86 degrees) compared to the TMTT with the CHLR group. A similar trend was observed in passive external rotation in adduction, with a 46-degree improvement in the TMTT with the CHLR group and a 34-degree improvement in the LDTT group. The LDTT group exhibited a lower preoperative function (15 degrees vs. 22 degrees) than the TMTT with the CHLR group, resulting in a correspondingly lower postoperative outcome (49 degrees vs. 68 degrees).

In BPBI cases involving upper roots, the transfer of the LD tendon alone or in conjunction with the TM tendon to the supraspinatus tendon, as recommended by Hoffer, 3 4 has traditionally been used to restore the balance of rotation forces. However, in most cases, the presence of subscapularis muscle contracture and retraction of the anterior coracohumeral ligament, combined with contracture of the TM muscle, results in scapula retraction, imposing limitations on abduction. 5 6 7 Therefore, we propose a surgical intervention consisting of two steps. First, the anterior contracture is released by sectioning the retracted anterior coracohumeral ligament. 8 9 Second, we perform the transfer of the TM tendon to the intersection between the supraspinatus and infraspinatus without disinserting the LD tendon, which continues to exert its function as an adductor and internal rotator. The primary objective of integrating both surgical techniques is to enable the active function of the shoulder musculature, ensuring both abduction and external rotation and therefore hand-mouth and hand–head movements.

The technique of anterior release of the shoulder through resection of the anterior coracohumeral ligament, as described by Sarac et al, 9 offers a method for partially restoring the balance of forces without necessitating intervention on the subscapularis muscle. Various techniques for posterior sliding of the subscapularis muscle to release internal rotation contractures exist, all of which are modifications of the original technique introduced by Gilbert et al, 10 who recommend its application only in cases displaying glenohumeral joint congruence. Some studies have described an anterior release using arthroscopic techniques, 11 12 which, if performed in the early stages, contribute to glenoid remodeling and improved humeral head coverage. However, the release of the subscapularis muscle from its insertion can lead to muscle damage and a recurrence of internal contracture in most cases, as described in the literature. 13 14 15 16 Therefore, our center refrains from performing subscapularis muscle sliding to prevent loss of internal rotation strength. Our surgical experience aligns with observations made by Sarac, indicating that anterior release through resection of the anterior coracohumeral ligament is a minimally invasive technique that enhances external rotation. Consequently, we have incorporated this technique into our new TMTT proposal, and our combined approach has demonstrated superior results.

Bahm proposes a stepwise anterior release algorithm with multiple steps, each performed based on the severity of the contracture. In addition to the initial sectioning of the anterior coracohumeral ligament, his approach involves resecting the coracoacromial ligament, total or subtotal coracoid process, and subscapularis tendon with an associated tenotomy-sliding. 17

Some authors have previously employed TM transfer to correct shoulder abduction by performing a pedicled unipolar transfer while maintaining the insertion of the joint tendon. This involves releasing its insertion at the inferior angle of the scapula and tunneling it under the proximal humerus to secure it over the clavicle or at the deltoid insertion. 18 However, a unipolar transfer of the TM to the rotator cuff at the intersection between the supraspinatus and infraspinatus, instead of detouring around the humeral neck, offers biomechanical advantages, providing greater correction of external rotation. 19 20 This improvement in global shoulder function is believed to be attributed to the so-called “force couple effect” 21 and the stabilization of the rotator cuff, which, to some extent, restores the mobility of the deltoid muscle.

To ensure the safe harvesting of the TM tendon, precise knowledge of its exact insertion footprint in the proximal humerus and its anatomical variations is crucial. 22 23 The TM inserts on the crest of the lesser tubercle, slightly medial and distal to the insertion of the LD in the bicipital groove, with a 2:1 ratio of insertional dimension relative to the latter. Although these muscles share a close anatomical relationship, their insertions are approximately 5 mm apart. Sixty percent of the proximal insertion of the TM is covered by the LD, and in 25% of cases, there is a connection between both tendons. Notably, in 50% of cases, there are fibers of considerable thickness in the proximal and medial part of the TM insertion, referred to as TM accessorius. Likewise, there is an additional belly in the distal part of the TM, described as a distal muscle slip of the TM, present in up to 75% of cases. 24 This structure is inserted separately but continuously and enveloped by the epimysium of the TM.

Our study has some inherent limitations due to the retrospective, single-center design, sample size, lack of randomization, and the inherent challenges associated with functional exploration in pediatric patients. Nevertheless, we have successfully demonstrated an effective and safe surgical alternative for tendon transfer in BPBI, yielding apparently superior results to the gold standard technique. Larger series with longer follow-up and prospective studies are required to confirm the validity of this technique.

## Conclusion

The transfer of the TM tendon to the supraspinatus tendon, combined with the release of the anterior coracohumeral ligament, results in a significant improvement in shoulder abduction and external rotation for patients with BPBI presenting internal rotation contracture. This approach emerges as a more favorable therapeutic option when compared to the transfer of the latissimus dorsi tendon to supraspinatus, showcasing superior functional outcomes.
